# Prognostic value of preoperative and postoperative serum CEA in colorectal signet ring cell carcinoma

**DOI:** 10.3389/fsurg.2025.1501436

**Published:** 2025-03-04

**Authors:** Yanan Zheng, Yang Luo, Zuhong Ji, Ying Pan, Xiaohong Wang, Fang Liu, Lei Liu, Shanshan Shen, Qiang You, Tao Ling

**Affiliations:** ^1^ Department of Geriatrics, Medical Center for Digestive Diseases, Second Affiliated Hospital of Nanjing Medical University, Nanjing, China; ^2^ Department of Nephrology, The Ninth People's Hospital of Chongqing, Chongqing, China; ^3^ Department of Gastroenterology, Nanjing Drum Tower Hospital Affiliated to Nanjing University Medical School, Nanjing, China; ^4^ Department of Gastroenterology, Xuzhou Central Hospital, Xuzhou, China; ^5^ Department of Gastroenterology, The Affiliated Yixing Hospital of Jiangsu University, Yixing, China; ^6^ Department of Critical Care Medicine, Jinhua Central Hospital, Jinhua, China; ^7^ Department of Gastroenterology, Jinhua Central Hospital, Jinhua, China

**Keywords:** carcinoembryonic antigen, prognosis, nomogram, colorecetal, signet ring cell carcinoma

## Abstract

**Background:**

Colorectal signet ring cell carcinoma (SRCC) is a rare and poorly prognosed tumor with limited established prognostic indicators. This study aims to investigate the prognostic value of serum carcinoembryonic antigen (CEA) in patients with colorectal SRCC.

**Methods:**

A retrospective, multicenter study was conducted to assess the association between CEA levels and survival outcomes in 942 patients with colorectal SRCC.

**Results:**

Patients exhibiting preoperative CEA (preCEA)-positivity demonstrated significantly lower cancer-specific survival (CSS) compared to those with preCEA-negativity in both Chinese and SEER datasets (5-year CSS: 27.50% vs. 48.27%, *P* = 0.01; 34.37% vs. 48.47%, *P* < 0.05). This disparity in outcomes was particularly notable in advanced stages (III, IV and N2; all *P* values < 0.05), while no statistical significance was observed in earlier stages (I/II, N0 and N1; all *P* values > 0.05). Patients with preCEA and postoperative CEA (postCEA)-negativity showed similar CSS to those with preCEA-positivity and postCEA-negativity, but those with postCEA-positivity had worse prognosis. After accounting for potential confounders, preCEA and postCEA maintained as independent predictors for CSS (*P* < 0.05). The nomogram model incorporating preCEA (preCEA-model) showed a C-index value of 0.75, whereas the model incorporating postCEA (postCEA-model) exhibited a C-index value of 0.73.

**Conclusions:**

Both preoperative and postoperative elevation of CEA levels were associated with adverse outcomes, with preoperative CEA demonstrating particularly significant predictive value in advanced-stage tumors. These findings propose that CEA could be a valuable tool for dynamically monitoring the prognosis of colorectal SRCC patients.

## Introduction

Signet ring cell carcinoma (SRCC) stands out as a rare and unique malignant tumor distinguished by the substantial accumulation of intracellular mucin, typically mucin 1 and 2, in over 50% of cells. Its characteristic presentation involves a crescent-shaped nucleus positioned eccentrically (1). Colorectal SRCC accounts for 15.3% of all SRCC cases and 1% of colorectal cancer (CRC) cases. In contrast to typical colorectal adenocarcinoma, colorectal SRCC frequently presents with regional or distant metastasis at the time of diagnosis, leading to a less favorable prognosis (1, 2). Due to the infrequent occurrence of the disease, research on the prognosis of SRCC is currently limited. The majority of existing studies are derived from single-center, small-sample studies, including few patients.

Carcinoembryonic antigen (CEA), initially identified in 1965 has been used as a serological tumor marker for CRC (3). Preoperative serum CEA (preCEA) levels impact overall survival (OS) and disease-free survival (DFS), serving as potential predictive indicators of cancer recurrence (4). Nevertheless, certain studies propose that postoperative serum CEA (postCEA) levels offer a more accurate prediction of recurrence, particularly within the first year after surgery (5).

Currently, the prognosis prediction for patients with signet ring cell carcinoma (SRCC) of the colorectum primarily depends on the TNM staging system, which lacks clinical markers that are suitable for dynamic assessment. Consequently, this study utilizes data from four Chinese hospitals and the United States SEER database to evaluate the prognostic value of preoperative and postoperative CEA levels in colorectal SRCC.

## Materials and methods

### Patients and study design

A retrospective multicenter study was conducted. The training set comprised 162 eligible patients diagnosed with colorectal SRCC from four tertiary hospitals in China between 2011 and 2020. The validation set consisted of 780 eligible patients extracted from the SEER database using SEER*Stat 8.4.0.1 between 2010 and 2019. All data collection personnel underwent standardized training and followed a unified Standard Operating Procedure (SOP) for data recording and verification. The inclusion criteria for this study encompassed patients with colorectal SRCC who had undergone surgical curative resection. Exclusion criteria included non-primary surgery, other concurrent malignancies, perioperative mortality, incomplete clinicopathological data, and the use of neoadjuvant therapy. The flow diagram is shown in Supplementary Figure 1. The study was conducted in accordance with Declaration of Helsinki and approved by the ethics committees of the four Chinese hospitals (Nanjing Medical University Second Affiliated Hospital, No. 2020-092; Yixing Hospital, No. 2022-158; Nanjing Gulou Hospital, No. 2022-469-02; Xuzhou Central Hospital, No. XZXY-LK-20240116-007).

### Study variables

The variables examined in this study encompassed age, gender, tumor site, tumor size, histological classification, TNM staging, tumor deposits (TD), perineural invasion (PNI), chemotherapy, preCEA and postCEA. In the Chinese dataset, preCEA referred to the CEA value measured before surgery, while postCEA represented the CEA value obtained within 3 months after surgery and before adjuvant chemotherapy (5). In cases where patients underwent multiple CEA tests during the period, the highest value was recorded. A CEA level exceeding 5 ng/ml was classified as positive, whereas levels ranging from 0 to 5 ng/ml were classified as negative. In the SEER dataset, CEA specifically referred to preoperative CEA. Survival outcomes for Chinese patients were garnered through proactive telephone-based follow-up and the mortality registration system. Survival data pertaining to the SEER database was acquired through established application procedures. CSS was measured from the date of cancer diagnosis to colorectal SRCC-related death, while OS was calculated as the time interval from diagnosis to death from any cause.

### Statistical analysis

Continuous variables and categorical variables were analyzed using Wilcoxon rank sum and chi-square tests, and were presented as medians and interquartile ranges [median (first quartile; third quartile)], as well as absolute values and percentages. Survival curves were constructed by Kaplan–Meier methodology and assessed using log-rank test. Univariate and multivariate survival analyses were conducted through the Cox proportional hazards model to minimize the impact of confounding factors on the study results. The hazard ratios (HR) accompanied by their corresponding 95% confidence intervals (CI) were calculated. A nomogram model was developed based on significant variables from the multivariate Cox model in the training set. The predictive ability of the model was assessed using the C-index, and its accuracy and calibration were validated through time-dependent ROC and calibration curves. All *P*-values were two-sided, with a significance level of *P* < 0.05 considered statistically significant. Data analysis was conducted using GraphPad Prism (version 9.4.1), SPSS 21.0 (SPSS, Chicago, IL), and R software (version 4.2.1).

## Results

### Basic patient characteristics

A total of 942 patients with colorectal SRCC were enrolled in the study. The 5-year CSS rates were 37.3% and 40.6% in the Chinese and SEER datasets, respectively, while the 5-year OS rates were 33.3% and 33.8%. In the Chinese dataset, 162 patients underwent preCEA testing, and 149 had postCEA testing. Among them, 80 patients (49.4%) showed preCEA-positivity, 53 (35.6%) exhibited postCEA-positivity, and 43 patients exhibited CEA-positivity both before and after surgery (Supplementary Table 1). Compared to those with negative CEA results, patients with both preCEA-positivity and postCEA-positivity were more likely to have larger tumors (diameter >5 cm) in the Chinese dataset (37.8% vs. 60.0%, *P* = 0.01; 39.6% vs. 67.9%, *P* = 0.001; Table 1). Meanwhile, individuals with preCEA-positivity in the SEER dataset were more frequently older, male, had larger tumors, were at T3 or T4 stage, exhibited N2 stage, M1 stage, tumor deposits, and were less likely to undergo chemotherapy (Table 1).

**Table 1:** Patient characteristics by preoperative CEA in the two sets.

Characteristic^a^ (*N*, %)	The Chinese dataset			The Chinese dataset			The SEER dataset		
	preCEA-negativity (*N* = 82)	preCEA-positivity (*N* = 80)	*P*^b^ value	postCEA-negativity (*N* = 96)	postCEA-positivity (*N* = 53)	*P*^b^ value	preCEA-negativity (*N* = 344)	preCEA-positivity (*N* = 436)	*P*^b^ value
Age			0.97			0.16			<0.01
Median (Q1–Q3)	60.0 (49.0–68.0)	59.5 (44.0–69.0)		58.5 (48.3–67.0)	62.0 (44.0–70.5)		63.0 (52.3–74.0)	69.0 (57.0–80.0)	
Gender			0.84			0.30			0.02
Female	51 (62.2)	51 (63.8)		57 (59.4)	36 (67.9)		184 (53.5)	197 (45.2)	
Male	31 (37.8)	29 (36.3)		39 (40.6)	17 (32.1)		160 (46.5)	239 (54.8)	
Tumor site			0.06			0.13			0.15
Right	23 (28.0)	15 (18.8)		24 (25.0)	12 (22.6)		229 (66.6)	316 (72.5)	
Left	27 (32.9)	19 (23.8)		31 (32.3)	10 (18.9)		102 (29.7)	110 (25.2)	
Rectum	32 (39.0)	46 (57.5)		41 (42.7)	31 (58.5)		13 (3.8)	10 (2.3)	
Tumor size (cm)			0.01			0.001			<0.01
≤5 cm	51 (62.2)	32 (40.0)		58 (60.4)	17 (32.1)		207 (60.2)	131 (30.0)	
>5 cm	31 (37.8)	48 (60.0)		38 (39.6)	36 (67.9)		137 (39.8)	305 (70.0)	
Grade			0.42			0.32			0.77
Moderate	4 (4.9)	2 (2.5)		5 (5.2)	1 (1.9)		19 (5.5)	22 (5.0)	
Poor	78 (95.1)	78 (97.5)		91 (94.8)	52 (98.1)		325 (94.5)	414 (95.0)	
T stage			0.30			0.24			<0.01
T1	1 (1.2)	0 (0.0)		1 (1.0)	0 (0.0)		12 (3.5)	3 (0.7)	
T2	4 (4.9)	1 (1.3)		4 (4.2)	0 (0.0)		21 (6.1)	10 (2.3)	
T3	39 (47.6)	34 (42.5)		45 (46.9)	21 (39.6)		158 (45.9)	184 (42.2)	
T4	38 (46.3)	45 (56.3)		46 (47.9)	32 (60.4)		153 (44.5)	239 (54.8)	
N stage			0.99			0.08			0.02
N0 (0 nodes)	14 (17.1)	14 (17.5)		21 (21.9)	4 (7.5)		75 (21.8)	82 (18.8)	
N1 (1–3 nodes)	15 (18.3)	14 (17.5)		16 (16.7)	11 (20.8)		78 (22.7)	71 (16.3)	
N2 (≥4 nodes)	53 (64.6)	52 (65.0)		59 (61.5)	38 (71.7)		191 (55.5)	283 (64.9)	
M			0.42			0.08			<0.01
M0	62 (75.6)	56 (70.0)		76 (79.2)	35 (66.0)		275 (79.9)	280 (64.2)	
M1	20 (24.4)	24 (30.0)		20 (20.8)	18 (34.0)		69 (20.1)	156 (35.8)	
TD			0.54			0.61			0.003
Absent	55 (67.1)	50 (62.5)		62 (64.6)	32 (60.4)		226 (65.7)	240 (55.0)	
Present	27 (32.9)	30 (37.5)		34 (35.4)	21 (39.6)		118 (34.3)	196 (45.0)	
Chemotherapy			0.78			0.15			0.01
No	26 (31.7)	27 (33.8)		27 (28.1)	21 (39.6)		120 (34.9)	195 (44.7)	
Yes	56 (68.3)	53 (66.3)		69 (71.9)	32 (60.4)		224 (65.1)	241 (55.3)	
PNI			0.62			0.50			0.80
Absent	46 (56.1)	48 (60.0)		58 (60.4)	29 (54.7)		231 (67.2)	289 (66.3)	
Present	36 (43.9)	32 (40.0)		38 (39.6)	24 (45.3)		113 (32.8)	147 (33.7)	

CEA, carcinoembryonic antigen; preCEA, preoperative CEA; postCEA, postoperative; SD, standard deviation; Q1-Q3, first quartile-third quartile; TD, tumor deposit; PNI, perineural invasion.

^a^ Continuous variables are presented in median(Q1–Q3), categorical variables are presented in counts (percentages).

^b^ Categorical variables were assessed by *χ*^2^ test, while continuous variables by the Wilcoxon rank-sum test.

### Prognostic assessment of serum CEA through Kaplan-Meier analysis

**Figure 1:**
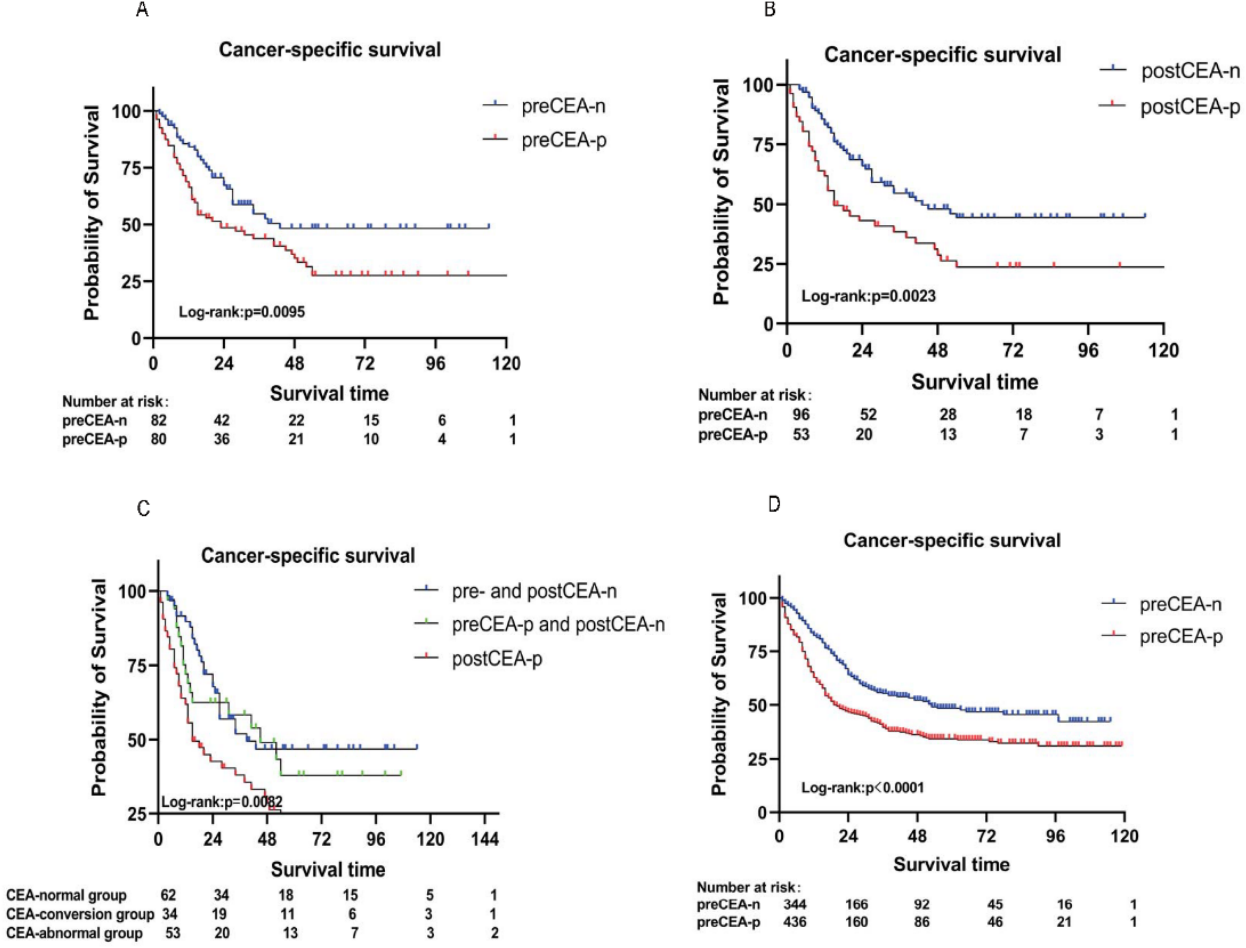
Kaplan–Meier analysis for CSS by preCEA **(A)**, postCEA **(B)**, and pre- and postCEA changes **(C)** in the Chinese dataset. Additionally, Kaplan–Meier analysis for CSS by preCEA in the SEER dataset **(D)** CSS, cancer-specific survival; CEA, carcinoembryonic antigen; preCEA-n, preoperative CEA-negativity; preCEA-p, preoperative CEA-positivity; postCEA-n, postoperative CEA-negativity; postCEA-p, postoperative CEA-positivity.

In the Chinese dataset, patients with preCEA-positivity exhibited significantly worse CSS compared to those with preCEA-negativity, with 5-year CSS rates of 27.50% vs. 48.27%, respectively (*P* = 0.01; Figure 1A). This disparity was particularly notable in stage III and N2 cases (all *P* values < 0.05; Supplementary Figures 2B,F), while lacking statistical significance in stages I/II, N0 and N1 (all *P* values > 0.05; Supplementary Figures 2A,D,E). In stage IV, the difference was not significant due to the smaller number of cases (*P* values >.05; Supplementary Figure 2C). The 5-year CSS rates for the postCEA-positivity and postCEA-negativity groups were 23.6% and 44.1% (*P* = 0.002; Figure 1B). Patients were classified into three groups based on the trend in CEA changes: pre- and postCEA-negativity group, preCEA-positivity and postCEA-negativity group, and postCEA-positivity group (Supplementary Table 1). Kaplan–Meier analysis revealed corresponding 5-year CSS rates of 46.86%, 38.09%, and 23.63%, respectively (*P* = 0.01; Figure 1C). Interestingly, the pre- and postCEA-negativity group exhibited a CSS comparable to the preCEA-positivity and postCEA-negativity group (HR, 1.23, 95% CI, 0.67–2.26, *P* = 0.51), both of which were superior to the postCEA-positivity group. In the SEER dataset, patients with preCEA-positivity had lower CSS compared to those with preCEA-negativity (5-year CSS: 34.37% vs. 48.47%, *P* < 0.05; Figure 1D). This trend was particularly notable in advanced stages (III, IV and N2; all *P* values < 0.05; Supplementary Figures 3B,C,F), whereas it lacked statistical significance in earlier stages (I/II, N0 and N1; all *P* values > 0.05; Supplementary Figures 3A,D,E).

### Univariate and multivariate analyses of serum CEA

Univariate Cox regression analysis revealed that T stage, N stage, M stage, TD, PNI, and preCEA were significantly associated with CSS in the Chinese dataset. After adjusting for covariates including T stage, N stage, M stage, TD, and PNI, the multivariate analysis demonstrated significant associations of preCEA with CSS (HR: 1.89, 95%CI:1.2–2.97, *P* = 0.01; Table 2). The prognostic value of preCEA remained consistent in the SEER database (Supplementary Table 2). After adjusting for covariates including T stage, N stage, M stage, TD, and PNI, postCEA remained an independent prognostic factor for CSS (HR: 1.76, 95%CI:1.11–2.80, *P* = 0.02; Table 3). Similar results were observed in the analysis of OS (Supplementary Tables 3–5).

**Table 2:** Univariate and multivariate analyses for cancer-specific survival based on preoperative CEA in the Chinese dataset.

Variables	Univariate^a^		Multivariate^b^	
	HR (95% CI)	*P* value	HR (95% CI)	*P* value
Age	1.00 (0.99–1.01)	0.87		
Gender				
Female				
Male	0.82 (0.52–1.28)	0.38		
Tumor site		0.94		
Right				
Left	0.92 (0.50–1.67)	0.78		
Rectum	0.91 (0.54–1.55)	0.73		
Tumor size				
≤5 cm				
>5 cm	1.09 (0.71–1.68)	0.68		
Grade				
Moderate				
Poor	2.60 (0.64–10.59)	0.18		
T stage				
T1–3				
T4	2.70 (1.71–4.29)	<0.01	1.73 (1.06–2.81)	0.03
N stage		0.01		
N1 (0 nodes)				0.18
N1 (1–3 nodes)	2.06 (0.82–5.17)	0.12	1.19 (0.45–3.15)	0.73
N2 (≥4 nodes)	3.36 (1.54–7.34)	0.002	1.83 (0.80–4.17)	0.15
M				
M0				
M1	2.83 (1.82–4.40)	<0.01	1.82 (1.10–3.00)	0.02
TD				
Absent				
Present	3.03 (1.96–4.69)	<0.01	1.85 (1.10–3.11)	0.02
Chemotherapy				
No				
Yes	1.27 (0.79–2.03)	0.32		
PNI				
Absent				
Present	2.25 (1.45–3.49)	<0.01	1.85 (1.14–3.02)	0.01
preCEA				
Negative				
Positive	1.76 (1.14–2.73)	0.01	1.89 (1.2–2.97)	0.01

HR, hazard ratio; CI, confidence interval; TD, tumor deposit; PNI, perineural invasion; CEA, carcinoembryonic antigen; preCEA, preoperative CEA.

^a^ Univariable Cox proportional hazards regression models.

^b^ Multivariable Cox proportional hazards regression model included T stage, N stage, M stage, TD, PNI, and preCEA.

**Table 3:** Univariate and multivariate analyses for cancer-specific survival based on postoperative CEA in the Chinese dataset.

Variables	Univariate^a^		Multivariate^b^	
	HR (95% CI)	*P* value	HR (95% CI)	*P* value
Age	1.00 (0.99–1.01)	0.87		
Gender		0.38		
Female	1 (Reference)			
Male	0.82 (0.52–1.28)			
Tumor site		0.94		
Right	1 (Reference)			
Left	0.92 (0.50–1.67)	0.78		
Rectum	0.91 (0.54–1.55)	0.73		
Tumor size				
≤5 cm	1 (Reference)			
>5 cm	1.09 (0.71–1.68)	0.68		
Grade				
Moderate	1 (Reference)			
Poor	2.60 (0.64–10.59)	0.18		
T stage				
T1–3				
T4	2.70 (1.71–4.29)	<0.01	1.81 (1.08–3.05)	0.03
N stage		0.01		0.22
N1 (0 nodes)	1 (Reference)			
N1 (1–3 nodes)	2.06 (0.82–5.17)	0.12	0.84 (0.31–2.30)	0.74
N2 (≥4 nodes)	3.36 (1.54–7.34)	0.002	1.44 (0.63–3.31)	0.39
M				
M0	1 (Reference)			
M1	2.83 (1.82–4.40)	<0.001	1.40 (0.82–2.37)	0.22
TD				
Absent	1 (Reference)			
Present	3.03 (1.96–4.69)	<0.001	2.06 (1.20–3.53)	0.01
Chemotherapy				
No	1(Reference)			
Yes	1.27 (0.79–2.03)	0.32		
PNI				
Absent	1 (Reference)			
Present	2.25 (1.45–3.49)	<0.001	1.74 (1.05–2.87)	0.03
postCEA				
Negative	1 (Reference)			
Positive	1.95 (1.25–3.04)	0.003	1.76 (1.11–2.80)	0.02

HR, hazard ratio; CI, confidence interval; TD, tumor deposit; PNI, perineural invasion; CEA, carcinoembryonic antigen; postCEA, postoperative CEA.

^a^ Univariable Cox proportional hazards regression models.

^b^ Multivariable Cox proportional hazards regression model included T stage, N stage, M stage, TD, PNI, and postCEA.

### Construction of the nomogram models

**Figure 2:**
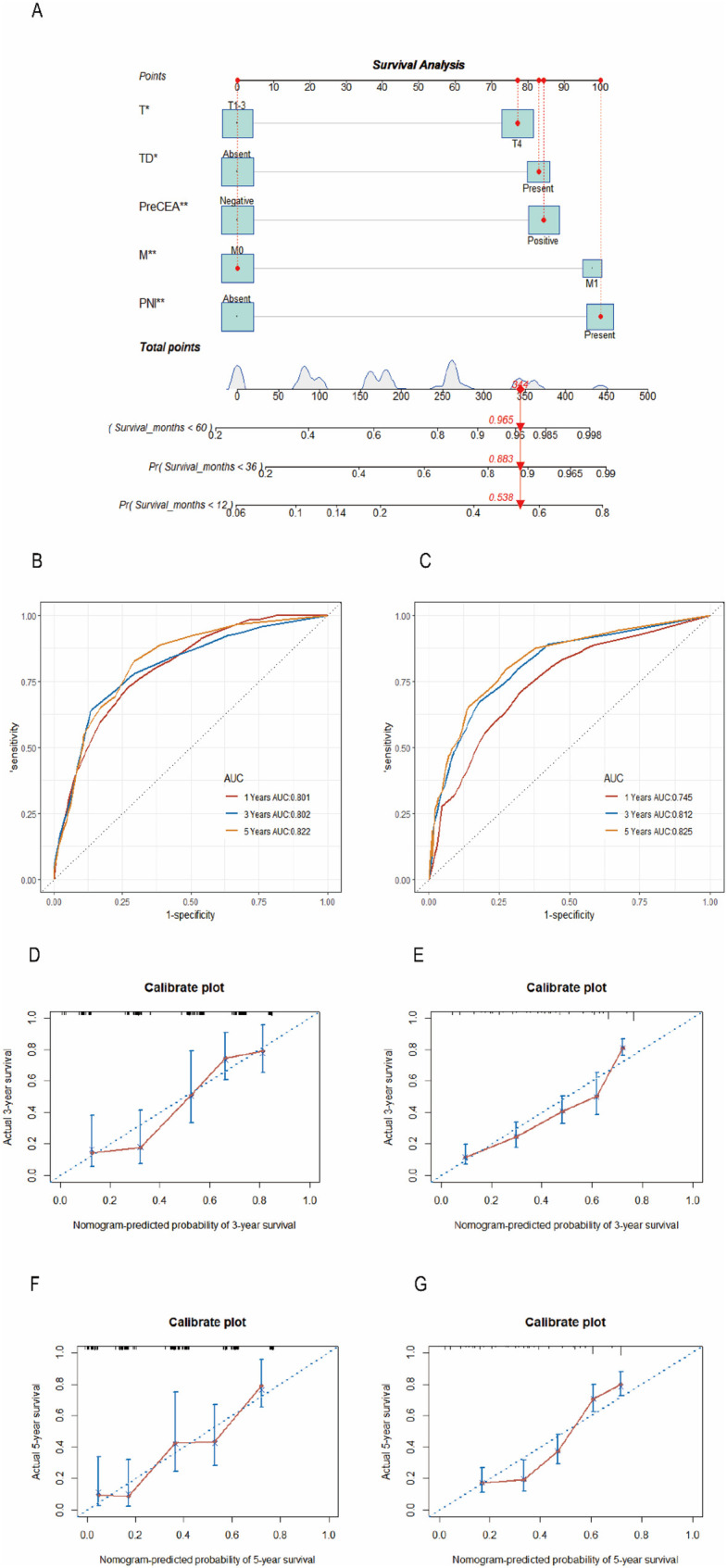
Nomogram of the CSS prognostic model incorporating preCEA in the Chinese dataset **(A)**, the time-dependent ROC **(B,C)**, the 3-year **(D,E)**, and 5-year calibration curves **(F,G)** were validated through internal and external validation. CSS, cancer-specific survival; CEA, carcinoembryonic antigen; preCEA, preoperative CEA; TD, tumor deposit; PNI, perineural invasion.

**Figure 3:**
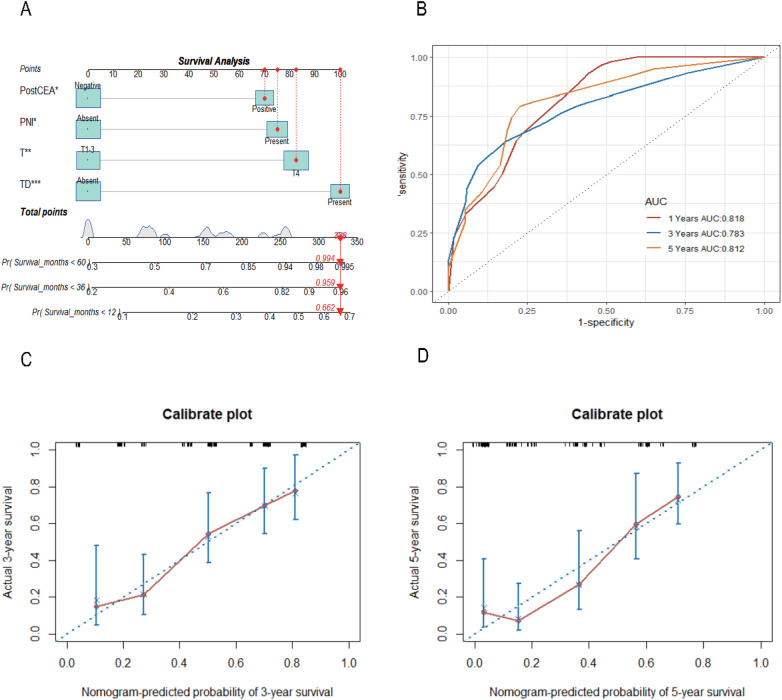
Nomogram of the CSS prognostic model incorporating postCEA in the Chinese dataset **(A)**, the time-dependent ROC **(B)**, the 3-year **(C)** and 5-year **(D)** calibration curves through internal validation. CSS, cancer-specific survival; CEA, carcinoembryonic antigen; postCEA, postoperative CEA; TD, tumor deposit; PNI, perineural invasion.

Utilizing the significant variables identified within the Chinese dataset, we constructed a nomogram model for predicting CSS incorporating preCEA (preCEA-model; Figure 2A). Internal validation revealed a C-index of 0.75 and AUC values of 0.80, 0.80, and 0.82 for predicting 1-year, 3-year, and 5-year CSS, respectively (Figure 2B). External validation using the SEER dataset showed a C-index of 0.71 and AUC values of 0.75, 0.81, and 0.83 for predicting 1-year, 3-year, and 5-year CSS, respectively (Figure 2C). In a subset of 149 cases with postCEA values in the Chinese dataset, a nomogram model incorporating postCEA (postCEA-model; Figure 3A) was developed. Internal validation resulted in a C-index of 0.73 and AUC values of 0.82, 0.78, and 0.81 for predicting 1-year, 3-year, and 5-year CSS, respectively (Figure 3B). The calibration curves indicated that both models exhibited good calibration for 3-year CSS and 5-year CSS (Figures 2D–G, 3C,D).

## Discussion

Colorectal SRCC was a rare histological subtype of adenocarcinoma, frequently associated with mutations in genes such as TP53, ARID1A, and APC (6) However, certain studies suggested that patients with colorectal SRCC had lower mutation rates in KRAS, PIK3CA, and APC compared to those with colorectal adenocarcinoma (7). Colorectal SRCC was typically diagnosed at advanced stages (stage III or IV), characterized by features such as a younger age, proximal tumor locations, and the presence of lymph node and peritoneal metastasis (8). Despite patients underwent curative surgery, postoperative recurrence remained common, resulting in a reduced overall survival period (2, 9). Patients with colorectal SRCC located on the left side and occurring at a younger age were associated with a poorer prognosis (10). It's worth noting that these insights were derived from studies with limited sample sizes, potentially impacting their generalizability. Consequently, it is crucial to dynamically assess prognosis using widely employed clinical markers.

CEA, an oncofetal glycoprotein associated with CRC (11), belonged to the CEA protein family, which was categorized into three groups: CEA cell adhesion molecules (CEACAM), pregnancy-specific glycoproteins (PSG), and pseudogenes (12). CEACAM, labeled as CD66a to CD66e, was linked to intracellular domains through transmembrane helices (13, 14). CEA demonstrated the capability to interact with dendritic cell-specific intercellular adhesion molecule-3-grabbing non-integrin (DC-SIGN), facilitating tumor adhesion and metastasis (15). Moreover, CEA inhibited cell differentiation through autocrine mechanisms, thereby providing support for the survival of tumor cells. Additionally, it promoted the development of endothelial cells and tumor blood vessels through paracrine signaling (16, 17). In multidrug-resistant metastatic CRC, elevated CEA expression had been associated with increased sensitivity to cetuximab, and inhibitors of the WNT/β*-*catenin pathway were found to enhance CEA expression. This implied the potential to augment the clinical efficacy of cetuximab by modulating CEA levels (18).

Preoperative CEA levels correlated with tumor staging and served as a predictive factor for CRC recurrence, often indicating a higher risk of mortality with a threshold of ≥5 ng/ml (19). However, a preoperative CEA level ≥2.1 ng/ml was also considered an adverse predictor for DFS in CRC patients (20). Preoperative CEA was recommended as a supplement to the TNM staging system (21). Nevertheless, some studies proposed that elevated preoperative CEA levels, which subsequently normalize following curative surgery, did not unequivocally signify an unfavorable prognosis (5). Postoperative elevation in serum CEA or tissue CEA levels was associated with a poorer prognosis in CRC, and the prognostic value of postoperative CEA appeared to surpass that of preoperative CEA (5, 22).

In the Chinese dataset of our study, 49.4% of cases exhibited preoperative CEA-positivity, while in the SEER dataset, this proportion was 55.9%, both surpassing the typically observed rates in colorectal adenocarcinoma, which ranged from 30% to 40% (5, 23). This variation may be associated with the poor differentiation of SRCC and its propensity for late-stage diagnosis, as these factors could potentially lead to increased CEA glycosylation. In addition, the prognostic significance of preoperative CEA was more pronounced in the later stages of the tumor (stage III, IV and N2), while exhibiting lower significance in the early stages (stage I/II, N0 and N1). This is consistent with previous literature, which identified preoperative CEA as an independent predictor for stages III-IV CRC (24).

The expression levels of CEA were correlated with the size of lung adenocarcinoma (25). Notably, our study found that patients exhibiting either preoperative CEA-positivity or postoperative CEA-positivity were associated with larger tumor diameters (often exceeding 5 cm). After adjusting for covariates in the multivariate model, both preoperative CEA and postoperative CEA retained their independent predictive significance for CSS and OS, consistent with the result in stages I-III colorectal cancer (26). Nomogram models based on CEA demonstrated excellent performance through internal and external validation. In comparison to the model incorporating preoperative CEA, the model involving postoperative CEA exhibited a lower C-index, potentially due to the extended time frame for postoperative CEA detection in the study. Nevertheless, dynamic monitoring of both preoperative and postoperative CEA remains clinically relevant for prognostic assessment in colorectal SRCC.

The strengths of this study lie in its utilization of a large, multicenter sample comprising 942 patients with colorectal SRCC to evaluate the prognostic significance of the tumor marker CEA in this uncommon malignancy. This extensive sample ensures a high level of statistical representativeness and enables generalization in the study of rare malignant tumors. Furthermore, rigorous statistical methods were applied and potential confounding factors were adequately addressed, thereby enhancing the reliability of our findings.

Despite possessing numerous significant advantages, the study inevitably has some limitations. Firstly, being a retrospective study, it may encounter risks of selection bias and information bias, potentially affecting the universal applicability of the conclusions. Secondly, the data for the study primarily originates from public databases and clinical records of four hospitals, raising concerns about the quality and completeness of the data.

Future research endeavors should be dedicated to conducting prospective cohort studies to further validate the prognostic significance of CEA in patients with colorectal SRCC. By implementing multi-center and cross-regional studies to expand the sample size, the aim is to enhance the generalizability of the research findings, thereby providing effective evaluations for precision treatment in cancer.

In conclusion, preoperative CEA can serve as a marker for assessing the prognosis of colorectal SRCC, particularly in advanced stages of the disease. Dynamic monitoring of both preoperative and postoperative CEA can provide new insights for clinical decision-making.

## Data Availability

The raw data supporting the conclusions of this article will be made available by the authors, without undue reservation.
