# Supplementary material for: Prognostic value of preoperative and postoperative serum CEA in colorectal signet ring cell carcinoma
Source: Front Surg. 2025 Mar 4;12:1501436. doi: 10.3389/fsurg.2025.1501436 (PMC11913837; doi:10.3389/fsurg.2025.1501436)
Supplement: Supplementary file 1 [file Supplementaryfile1.docx]

**Supplementary Figures**

Figure S1
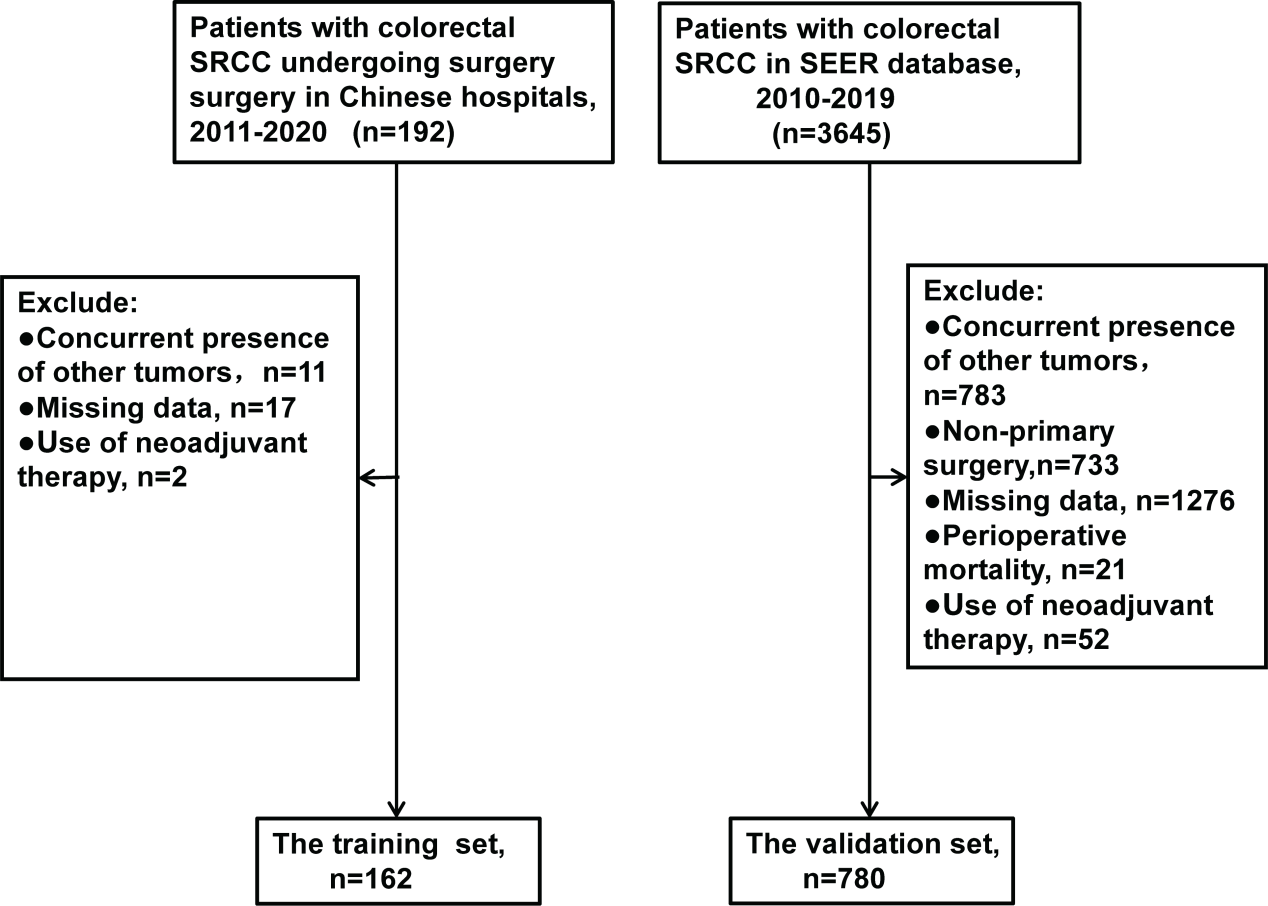


Supplementary Figure 1 The flowchart for this study

Figure S2


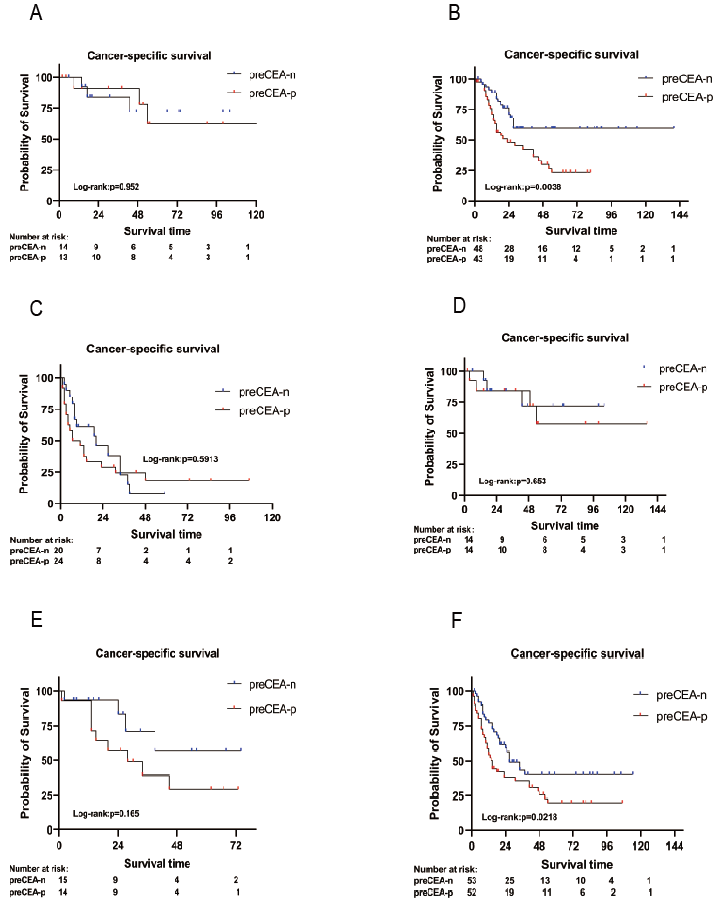


Supplementary Figure 2 Kaplan-Meier analysis for CSS by preCEA in stage I/II (A), III (B), IV (C), N0 (D), N1 (E), and N2 (F) in the Chinese dataset.

Figure S3


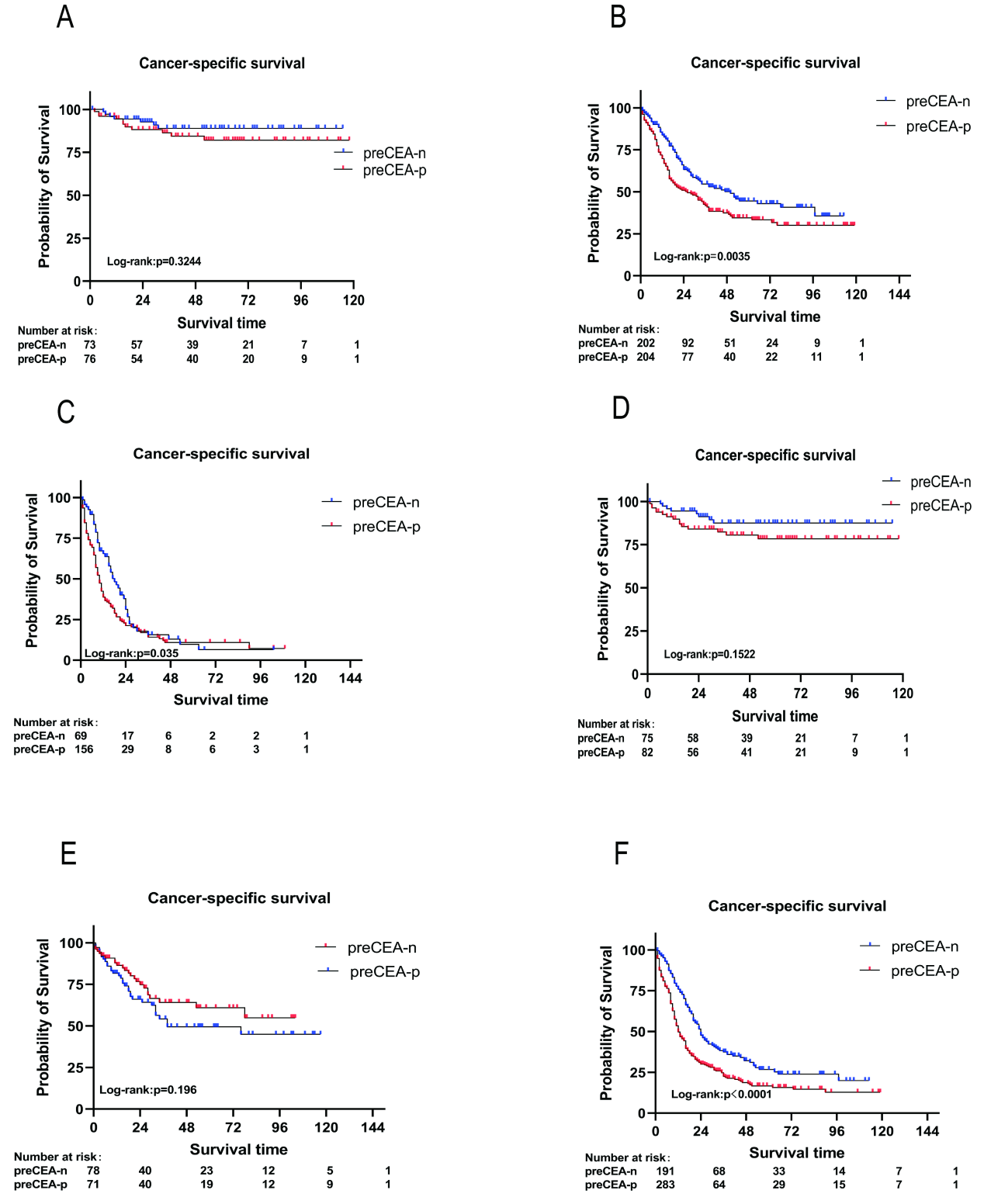


Supplementary Figure 3 Kaplan-Meier analysis for CSS by preCEA in stage I/II (A), III (B), IV (C), N0 (D), N1 (E), N2 (F) in the SEER dataset.
