# Supplementary material for: Prognostic value of preoperative and postoperative serum CEA in colorectal signet ring cell carcinoma
Source: Front Surg. 2025 Mar 4;12:1501436. doi: 10.3389/fsurg.2025.1501436 (PMC11913837; doi:10.3389/fsurg.2025.1501436)
Supplement: Supplementary file 2 [file Table1.docx]

**Supplementary Tables**

Supplyment Table 1. Preoperative and postoperative CEA changes in the Chinese dataset

|  | preCEA-negativity  N=82 | preCEA-positivity  N=80 | postCEA-negativity  N=96 | postCEA-positivity  N=53 |
| --- | --- | --- | --- | --- |
| pre-and postCEA-negativity  N=62 | 62 | NA | 62 | NA |
| preCEA-positivity/postCEA-  negativity N=34 | NA | 34 | 34 | NA |
| postCEA-positivity  N=53 | 10 | 43 | NA | 53 |
| CEA, carcinoembryonic antigen; preCEA, preoperative CEA; postCEA, postoperative CEA; NA, not applicable. | | | | |

Supplyment Table 2. Univariate and multivariate analyses for cancer-specific survival based on preoperative CEA in the SEER set

| Variables | Univariate^a^ | | Multivariate^b^ | |
| --- | --- | --- | --- | --- |
|  | HR (95% CI) | P value | HR (95% CI) | P value |
| Age | 1.00(1.00-1.01) | 0.68 |  |  |
| Gender |  |  |  |  |
| Female |  |  |  |  |
| Male | 0.93(0.77-1.14) | 0.49 |  |  |
| Tumor site |  | 0.82 |  |  |
| Right |  |  |  |  |
| Left | 1.07(0.86-1.33) | 0.57 |  |  |
| Rectum | 0.94(0.52-1.73) | 0.85 |  |  |
| Tumor size |  |  |  |  |
| ≤5cm |  |  |  |  |
| >5cm | 1.40(1.15-1.72) | 0.001 | 1.21(0.98-1.50) | 0.08 |
| Grade |  |  |  |  |
| Moderate |  |  |  |  |
| Poor | 1.22(0.77-1.93) | 0.41 |  |  |
| T stage |  |  |  |  |
| T1-3 |  |  |  |  |
| T4 | 2.76(2.24-3.41) | <0.01 | 1.60(1.28-2.00) | <0.01 |
| N stage |  | <0.01 |  | <0.01 |
| N1(0 nodes) |  |  |  |  |
| N1(1-3 nodes) | 3.17(1.95-5.16) | <0.01 | 2.51(1.53-4.12) | <0.01 |
| N2(≥4 nodes) | 8.06(5.26-12.33) | <0.01 | 4.80(3.05-7.57) | <0.01 |
| M |  |  |  |  |
| M0 |  |  |  |  |
| M1 | 3.43(2.79-4.20) | <0.01 | 1.78(1.42-2.23) | <0.01 |
| TD |  |  |  |  |
| Absent |  |  |  |  |
| Present | 2.74(2.24-3.36) | <0.01 | 1.29(1.03-1.61) | 0.03 |
| Chemotherapy |  |  |  |  |
| No |  |  |  |  |
| Yes | 1.02(0.83-1.26) | 0.84 |  |  |
| PNI |  |  |  |  |
| Absent |  |  |  |  |
| Present | 1.98(1.62-2.42) | <0.01 | 1.03(0.83-1.27) | 0.81 |
| preCEA |  |  |  |  |
| Negative |  |  |  |  |
| Positive | 1.69(1.38-2.08) | <0.01 | 1.47(1.19-1.82) | <0.01 |
| ^a^ Univariable Cox proportional hazards regression models. | | | | |
| ^b^ Multivariable Cox proportional hazards regression model included tumor size, T stage, N stage, M stage, TD, PNI, and preCEA. | | | | |
| HR, hazard ratio; CI, confidence interval; TD, tumor deposit; PNI, perineural invasion; CEA, carcinoembryonic antigen; preCEA, preoperative CEA. | | | | |

Supplyment Table 3. Univariate and multivariate analyses for overall survival based on preoperative CEA in the Chinese dataset

| Variables | | Univariate^a^ | | Multivariate^b^ | | |
| --- | --- | --- | --- | --- | --- | --- |
|  |  | HR (95% CI) | P value | HR (95% CI) | P value | |
| Age | | 1.01(0.99-1.02) | 0.405 |  |  | |
| Gender | |  |  |  |  | |
| Female | |  |  |  |  | |
| Male | | 0.90(0.59-1.37) | 0.61 |  |  | |
| Tumor site | |  | 0.93 |  |  | |
| Right | |  |  |  |  | |
| Left | | 0.90(0.52-1.58) | 0.72 |  |  | |
| Rectum | | 0.93(0.57-1.52) | 0.77 |  |  | |
| Tumor size | |  |  |  |  | |
| ≤5cm | |  |  |  |  | |
| >5cm | | 1.14(0.76-1.71) | 0.51 |  |  | |
| Grade | |  |  |  |  | |
| Moderate | |  |  |  |  | |
| Poor | | 2.94(0.72-11.97) | 0.13 |  |  | |
| T stage | |  |  |  |  | |
| T1-3 | |  |  |  |  | |
| T4 | | 2.26(1.48-3.45) | <0.01 | 1.42(0.91-2.22) | 0.12 | |
| N stage | |  | 0.01 |  | 0.15 | |
| N1(0 nodes) | |  |  |  |  | |
| N1(1-3 nodes) | | 3.12(1.31-7.43) | 0.01 | 1.902(0.758-4.772) | 0.17 | |
| N2(≥4 nodes) | | 3.66(1.68-7.97) | 0.001 | 2.21(0.98-5.00) | 0.06 | |
| M | |  |  |  |  | |
| M0 | |  |  |  |  | |
| M1 | | 2.56(1.68-3.90) | <0.01 | 1.56(0.97-2.5) | 0.07 | |
| TD | |  |  |  |  | |
| Absent | |  |  |  |  | |
| Present | | 3.22(2.13-4.87) | <0.01 | 2.09(1.29-3.38) | 0.003 | |
| Chemotherapy | |  |  |  |  | |
| No | |  |  |  |  | |
| Yes | | 1.06(0.69-1.62) | 0.80 |  |  | |
| PNI | |  |  |  |  | |
| Absent | |  |  |  |  | |
| Present | | 1.97(1.30-2.97) | 0.001 | 1.69(1.07-2.67) | 0.02 | |
| preCEA | |  |  |  |  | |
| Negative | |  |  |  |  | |
| Positive | | 1.72(1.13-2.59) | 0.01 | 1.80(1.18-2.74) | 0.01 | |
| ^a^ Univariable Cox proportional hazards regression models. | | | | |  |  |
| ^b^ Multivariable Cox proportional hazards regression model included T stage, N stage, M stage, TD, PNI, and preCEA. | | | | |  |  |
| HR, hazard ratio; CI, confidence interval; TD, tumor deposit; PNI, perineural invasion; CEA, carcinoembryonic antigen; preCEA, preoperative CEA. | | | | |  |  |

Supplyment Table 4. Univariate and multivariate analyses for overall survival based on postoperative CEA in the Chinese dataset

| Variables | | Univariate^a^ | | Multivariate^b^ | | |
| --- | --- | --- | --- | --- | --- | --- |
|  |  | HR (95% CI) | P value | HR (95% CI) | P value | |
| Age | | 1.01(0.99-1.02) | 0.41 |  |  | |
| Gender | |  |  |  |  | |
| Female | |  |  |  |  | |
| Male | | 0.90(0.59-1.37) | 0.61 |  |  | |
| Tumor site | |  | 0.93 |  |  | |
| Right | |  |  |  |  | |
| Left | | 0.90(0.52-1.58) | 0.72 |  |  | |
| Rectum | | 0.93(0.57-1.52) | 0.77 |  |  | |
| Tumor size | |  |  |  |  | |
| ≤5cm | |  |  |  |  | |
| >5cm | | 1.14(0.76-1.71) | 0.51 |  |  | |
| Grade | |  |  |  |  | |
| Moderate | |  |  |  |  | |
| Poor | | 2.94(0.72-11.97) | 0.13 |  |  | |
| T stage | |  |  |  |  | |
| T1-3 | |  |  |  |  | |
| T4 | | 2.26(1.48-3.45) | <0.01 | 1.45(0.91-2.33) | 0.12 | |
| N stage | |  | 0.01 |  | 0.3 | |
| N1(0 nodes) | |  |  |  |  | |
| N1(1-3 nodes) | | 3.12(1.31-7.43) | 0.01 | 1.39(0.54-3.58) | 0.49 | |
| N2(≥4 nodes) | | 3.66(1.68-7.97) | 0.001 | 1.78(0.78-4.05) | 0.17 | |
| M | |  |  |  |  | |
| M0 | |  |  |  |  | |
| M1 | | 2.56(1.68-3.90) | <0.01 | 1.25(0.76-2.06) | 0.38 | |
| TD | |  |  |  |  | |
| Absent | |  |  |  |  | |
| Present | | 3.22(2.13-4.87) | <0.01 | 2.28(1.38-3.75) | 0.001 | |
| Chemotherapy | |  |  |  |  | |
| No | |  |  |  |  | |
| Yes | | 1.06(0.69-1.62) | 0.80 |  |  | |
| PNI | |  |  |  |  | |
| Absent | |  |  |  |  | |
| Present | | 1.97(1.30-2.97) | 0.001 | 1.61(1.01-2.58) | 0.05 | |
| postCEA | |  |  |  |  | |
| Negative | |  |  |  |  | |
| Positive | | 2.03(1.34-3.07) | 0.001 | 1.81(1.18-2.78) | 0.01 | |
| ^a^ Univariable Cox proportional hazards regression models. | | | | |  |  |
| ^b^ Multivariable Cox proportional hazards regression model included T stage, N stage, M stage, TD, PNI, and postCEA. | | | | |  |  |
| HR, hazard ratio; CI, confidence interval; TD, tumor deposit; PNI, perineural invasion; CEA, carcinoembryonic antigen; postCEA, postoperative CEA. | | | | |  |  |

Supplyment Table 5. Univariate and multivariate analyses for overall survival based on preoperative CEA in the SEER set

| Variables | Univariate^a^ | | Multivariate^b^ | | |
| --- | --- | --- | --- | --- | --- |
|  | HR (95% CI) | P value | HR (95% CI) | P value | |
| Age | 1.01(1.00-1.02) | 0.005 | 1.02(1.01-1.03) | <0.01 | |
| Gender |  |  |  |  | |
| Female |  |  |  |  | |
| Male | 0.98(0.82-1.18) | 0.84 |  |  | |
| Tumor site |  | 0.94 |  |  | |
| Right |  |  |  |  | |
| Left | 0.99(0.80-1.21) | 0.90 |  |  | |
| Rectum | 0.91(0.52-1.58) | 0.73 |  |  | |
| Tumor size |  |  |  |  | |
| ≤5cm |  |  |  |  | |
| >5cm | 1.37(1.14-1.65) | 0.001 | 1.25(1.03-1.52) | 0.03 | |
| Grade |  |  |  |  | |
| Moderate |  |  |  |  | |
| Poor | 1.31(0.85-2.03) | 0.23 |  |  | |
| T stage |  |  |  |  | |
| T1-2 |  |  |  |  | |
| T3-4 | 3.77(2.01-7.05) | <0.01 | 1.29(0.67-2.48) | 0.45 | |
| N stage |  | <0.01 |  | <0.01 | |
| N1(0 nodes) |  |  |  |  | |
| N1(1-3 nodes) | 2.14(1.47-3.13) | <0.01 | 1.84(1.25-2.72) | 0.002 | |
| N2(≥4 nodes) | 4.85(3.54-6.66) | <0.01 | 3.71(2.61-5.29) | <0.01 | |
| M |  |  |  |  | |
| M0 |  |  |  |  | |
| M1 | 2.94(2.43-3.55) | <0.01 | 1.96(1.59-2.43) | <0.01 | |
| TD |  |  |  |  | |
| Absent |  |  |  |  | |
| Present | 2.44(2.03-2.94) | <0.01 | 1.39(1.12-1.73) | 0.003 | |
| Chemotherapy |  |  |  |  | |
| No |  |  |  |  | |
| Yes | 0.85(0.71-1.02) | 0.08 |  |  | |
| PNI |  |  |  |  | |
| Absent |  |  |  |  | |
| Present | 1.81(1.50-2.18) | <0.01 | 1.07(0.87-1.31) | 0.52 | |
| preCEA |  |  |  |  | |
| Negative |  |  |  |  | |
| Positive | 1.63(1.35-1.96) | <0.01 | 1.25(1.02-1.53) | 0.029 | |
| ^a^ Univariable Cox proportional hazards regression models. | | | | | |
| ^b^ Multivariable Cox proportional hazards regression model included age, tumor size, T stage, N stage, M stage, TD, PNI, and preCEA. | | | | | |
| HR, hazard ratio; CI, confidence interval; TD, tumor deposit; PNI, perineural invasion; CEA, carcinoembryonic antigen; preCEA, preoperative CEA. | | | | | |
